# Management of sialorrhea in children: a systematic review

**DOI:** 10.1590/1806-9282.20230971

**Published:** 2024-04-22

**Authors:** Manuela Leitão de Vasconcelos, Divany Guedes Pereira da Cunha, Giorvan Ânderson dos Santos Alves, Tatiana Carneiro da Cunha Almeida Santos, Luiz Medeiros de Araujo Lima-Filho, Karinna Veríssimo Meira Taveira, Cristiano Miranda de Araújo, Leandro Pernambuco

**Affiliations:** 1 Universidade Federal da Paraíba, Graduate Program in Decision Models and Health – João Pessoa (PB), Brazil.; 2 Universidade Federal da Paraíba, Lauro Wanderley University Hospital – João Pessoa (PB), Brazil.; 3 Universidade Federal da Paraíba, Department of Speech, Language and Hearing Sciences – João Pessoa (PB), Brazil.; 4 Universidade Federal da Paraíba, Department of Statistic – João Pessoa (PB), Brazil.; 5 Universidade Federal do Rio Grande Do Norte, Center for Advanced Studies in Systematic Review and Meta-analysis – Natal (RN), Brazil.; 6 Universidade Tuiuti do Paraná, Center for Advanced Studies in Systematic Review and Meta-analysis – Natal (RN), Brazil.

## BACKGROUND

Sialorrhea is an involuntary loss of saliva through the mouth, considered pathological in children aged 4 years and above, which may be due to increased saliva production or swallowing deficit, the latter being the most frequent condition in children with neurological disorders^2–5^.

This non-intentional saliva loss may happen anteriorly or posteriorly^3,4,6^. A child may have both types, with impacts on various dimensions of their and their caregivers’ lives. The literature describes health, emotional, and social impacts^2,5,7–9^.

The occurrence of sialorrhea ranges from 10 to 83%, with a higher frequency in children with neurological disorders^2,7,8^.

The literature points out different intervention methods. It indicates beginning sialorrhea management with less invasive strategies, then progressing to more invasive ones if the patient does not adequately respond to the treatment^8,10^.

Less invasive interventions aim to improve swallowing efficiency and frequency, decreasing saliva accumulation in the oral cavity^8,10,11^. Pharmacological therapy administers drugs to decrease saliva production, but it may have side effects such as urine retention and headaches^12^. Botulinum toxin has been used as an alternative to minimize these effects, which is usually applied to the glands that produce the greatest volume of unstimulated saliva. Surgery is the most invasive sialorrhea management strategy, ranging from salivary duct relocation to gland resection^13^.

Given the impact this condition may have on children's and caregivers’ lives, studies aim to assess the effectiveness of therapies to control sialorrhea^7,8,10,11,13^. Thus, the objective of this review was to analyze the efficacy of interventions to control sialorrhea in children.

## METHODS

This systematic review was conducted according to PRISMA 2020^14^. Eligibility criteria were established with PICOS and included research on treatments to control sialorrhea in children. No study was excluded based on language, time of publication, population sex, or ethnicity. Randomized clinical trials approaching sialorrhea control interventions in children aged up to 12 years were included. The exclusion criteria were as follows: 1. studies on therapeutic interventions including children aged above 12 years, without the possibility of distinguishing the specific results of the age group of interest for this review; 2. studies with results of sialorrhea control without a specific sialorrhea control intervention; 3. studies different from clinical trials; and 4. unavailable full-text articles.

Five databases were searched: Excerpta Medica database (EMBASE), Latin-American and Caribbean Health Sciences Literature (LILACS), PubMed/Medline, Scopus, and Web of Science. An additional search was made on the gray literature: Google Scholar, OpenGrey, ProQuest, and the Brazilian Digital Library of Theses and Dissertations, besides a manual search in the references to the articles included in the review. References were organized, and duplicates were removed using the EndNote^®^ online version^15^. The search took place on March 1, 2021, and was updated on January 16, 2022.

Two independent reviewers conducted the selection steps. All divergences regarding study selection were solved by a third reviewer. The kappa coefficient of agreement between reviewers was 0.7, indicating good agreement^16^.

The risk of bias was assessed with the Cochrane Collaboration Risk of Bias Tool^17^, and a chart was generated with the RevMan 5.4 software^18^. The difference between before and after the intervention was calculated. The mean difference was calculated for discrete quantitative variables, whereas median variation or percentage frequency was observed for the qualitative variables. The certainty of the evidence was assessed with the Grading of Recommendations, Assessment, Development, and Evaluation^19^.

## RESULTS

**Figure 1:**
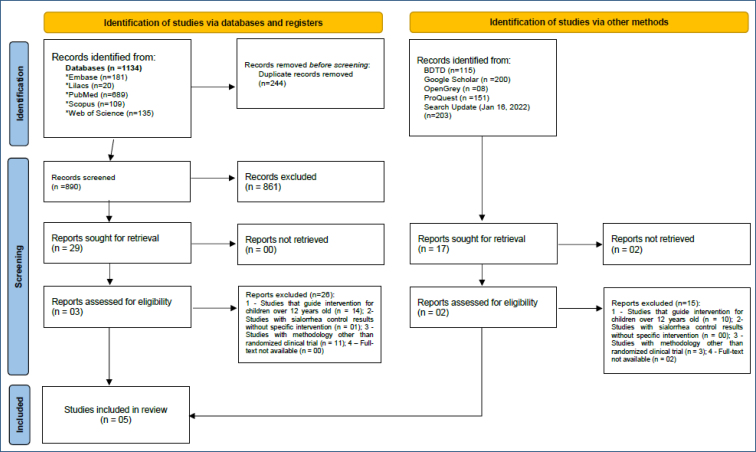
Flowchart of literature research and selection criteria. Source: Manuela Leitão de Vasconcelos.

The initial search found 1,608 articles. After analysis according to the eligibility criteria, five articles comprised the final sample of the qualitative synthesis (Figure 1).

The five articles included in the research were published between 2009 and 2019^7–11^. Their sample ranged from 24 to 53 subjects, aged 21 months to 12 years, all of them with neurological disorders.

The following sialorrhea control interventions were approached: behavioral therapy^11^, oral therapy motor exercises^8,10,11^, chewing training^10^, kinesio taping^8^, botulinum toxin^7,13^, and submandibular duct surgery^13^. The efficacy of these interventions was analyzed by comparing them with a placebo group or another type of intervention, assessed with the Drooling Severity and Frequency Scale^7,8,10,13^, Drooling Quotient^13^, Drooling Impact Scale^8^, visual analog scale^13^, and sialorrhea episode count^11^.

The articles used different instruments and measures to assess intervention efficacy. Moreover, different interventions were used, and therefore they could not be grouped. The description of article characteristics included in the review is shown in Table 1.

**Table 1:** Summary of descriptive characteristics of the studies included in the review (n=5).

Authors, Years, Countries	Objectives	Samples	Interventions	Effect Measures						Conclusion
				A			B			
				I	F	Difference (p-value)	I	F	Difference (p-value)	
Alrefai et al.^7^ Jordan	To investigate the efficacy and safety of injecting neurotoxin serotype A into the parotid glands to treat sialorrhea in children with cerebral palsy	Age: 21 months to 7 years S_I:_24 S_F_16	A: Placebo B: Botulinum toxin	**Sialorrhea Frequency Scale (median)**						Botulinum toxin proved to be an effective and safe option in sialorrhea treatment. No intergroup comparison results were presented. The experimental group decreased 2 points in the median, while the placebo group had no variation.
				4	4	0	4	3	1 (p=0.034)	
				**Sialorrhea Severity Scale (median)**						
				5	5	0	5	4	1 (p=0.026)	
				**Sialorrhea Total Score (median)**						
				9	9	0	9	7	2 (p=0.027)	
Awan et al.^8^ Pakistan	To determine the efficacy of KT in combination with OME to improve sialorrhea in children with cerebral palsy	Age: 4–8 years S_I:_ 48 S_F:_48	A: KT + OME B: OME	**Sialorrhea Frequency Scale (mean)**						Both groups had a significant decrease in the sialorrhea scale. In the frequency scale, group A had a final mean 0.34 lower than group B; in the severity scale, the final mean was 0.1 lower. The intergroup comparison had no statistical significance.
				3.86	2.30	1.56 (p=0.00)	3.88	2.64	1.24 (p=0.00)	
				**Sialorrhea Severity Scale (mean)**						
				4.00	2.47	1.53 (p=0.00)	3.25	2.48	0.77 (p=0.00)	
Bekkers et al.^13^ The Netherlands	To compare the effects of submandibular duct surgery with botulinum toxin application into the submandibular glands in children with neurodevelopmental disorders	Age: 11 years S_I:_ 57 S_F:_ 53	A: Botulinum toxin B: Submandibular gland duct surgery	**Visual Analog Scale (mean)**						Intergroup comparison had a significant difference in VAS outcomes (p<0.001); the surgical procedure had better sialorrhea results than the botulinum toxin.
				82.1	75.0	7.1	77	45.6	31.4	
				**Drooling Quotient (mean)**						
				28.7	24.8	3.9	26	15	11	
Inal et al.^10^ Turkey	To examine the effects of FuCT on tongue projection and sialorrhea in children with cerebral palsy	Age: 4–6 years S_I:_ 40 S_F:_32	A: FuCT B: Conventional exercises	**Sialorrhea Severity Scale (absolute number and percentage frequency)**						No significant decrease was observed regarding severity (p=0.210) and frequency (p=0.162) in the comparison of the two groups.
				Dry: 0 (0)	0 (0)	p=0.002	0 (0)	0 (0)	p=0.157	
				Mild: 0 (0)	4 (25)		2 (12.5)	3 (18.8)		
				Moderate: 6 (37.5)	8 (50)		5 (31.3)	5 (31.3)		
				Severity: 6 (37.5)	3 (18.8)		5 (31.3)	4 (25)		
				Profuse: 4 (25)	1 (6.3)		4 (25)	4 (25)		
				**Sialorrhea Frequency Scale (absolute number and percentage srequency)**						
				Never: 0 (0)	0 (0)	p=0.082	0 (0)	0 (0)	p=0.317	
				Occasionally: 3 (18.8)	5 (25)		1 (6.3)	1 (18.8)		
				Frequently: 7 (43.8)	8 (50)		9 (56.3)	10 (31.3)		
				Constantly: 6 (37.5)	3 (18.8)		6 (37.5)	5 (37.5)		
Sethy and Mokashi^11^ India	To investigate the efficacy of reward behavioral therapy in combination with conventional therapy to control sialorrhea in children with cerebral palsy associated with mild intellectual deficit	Age: 5–12 years S_I:_25 S_F:_25	A: Behavioral therapy in combination with conventional therapy B: Conventional therapy	**Sialorrhea Frequency**						Behavioral therapy in combination with conventional therapy had better sialorrhea frequency results, with a final mean 15.71 points lower than the conventional therapy group. However, no intergroup comparison result was presented.
				22.17	5.67	16.5 (p=0.001)	21.85	21.38	0.47 (p=0.070)	

A: group A; B: group B; I: initial measure; F: final measure; S_I_: initial sample; S_F_: final sample; KT: kinesio taping; OME: oral motor exercises; FuCT: functional chewing training; DQ: Drooling Quotient; VAS: visual analog scale. Source: Manuela Leitão de Vasconcelos.

**Figure 2:**
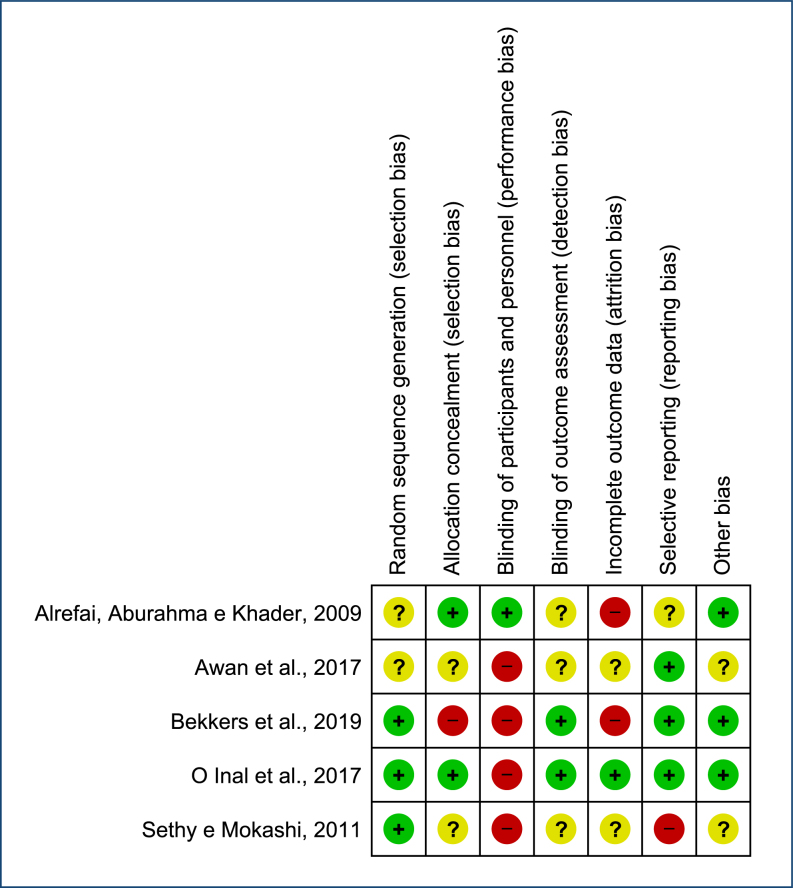
Assessment of the risk of bias in the studies included in the synthesis, assessed with ROB1. *Studies were assessed with ROB1. Green indicates a low risk of bias, yellow indicates an unclear risk, and red indicates a high risk of bias. Source: Manuela Leitão de Vasconcelos.

None of the articles met all the methodological quality criteria. The articles that reported random sequence generation^10,11,13^ used strategies such as draws and software. Only one article clearly stated the blinding of participants and personnel^7^. Three pieces of research did not present enough information on the blinding of outcome assessment^7,8,11^, while two were classified as low risk^10,13^. Regarding incomplete outcome data, two studies were classified as high risk^7,13^ because of frequent losses, which were unbalanced between the groups; two studies did not present enough information^8,11^; and one was classified as low risk^10^. Concerning selective reporting, one article did not make clear which outcomes would be assessed, characterizing high risk^11^, one study did not present enough information to assess^8^, and three were classified as low risk^7,10,13^ (Figure 2).

Given the few articles included in the analysis, publication bias could not be assessed with a funnel plot. However, the inclusion of LILACS with languages other than English, the broad search strategy, and the search in the gray literature diminish the likelihood of such bias occurring.

The certainty of the evidence of the following outcomes was assessed: total score of sialorrhea frequency and severity^7^; sialorrhea frequency^7,8,10^; sialorrhea severity^7,8,10^; sialorrhea episode count^11^; Drooling Quotient^13^; and Drooling Impact Scale^13^. They were classified as very low (the frequency and the severity of sialorrhea), low (sialorrhea episode count), and moderate (the total score of the sialorrhea frequency and severity scale, Drooling Quotient, and the impact of sialorrhea).

## DISCUSSION

This review investigated the efficacy of different intervention methods to manage sialorrhea in children. Treatment efficacy was assessed by comparing before and after intervention with different assessment instruments. Although all of them were compared before and after the interventions, only three articles presented comparisons between groups^8,10,13^, which reflected a risk of bias and the quality of evidence.

Sialorrhea assessment instruments are useful to diagnose it, define therapy procedures, and monitor interventions. The literature describes various assessment instruments for the general population^3^. However, the assessment of children, especially those with neurological disorders, must consider their skills before choosing which instrument will be used, since for some methods, it is necessary for the child to spend a period without swallowing as well as knowing how to spit.

The Drooling Severity and Frequency Scale was the most often used instrument to assess sialorrhea^20^, using which the examiner and/or caregiver directly observe and classify the saliva according to its frequency and severity. This instrument is important because it considers the circadian variation and the interference of factors such as hunger, thirst, fatigue, anxiety, and oral infections^3^.

Sialorrhea management interventions included in this review sample range from behavior-based strategies to surgical interventions^11,13^. The literature indicates that interventions must begin with less invasive methods and progress toward more invasive ones if children do not respond to the treatment^8,10^. The least invasive therapeutic strategies include speech and language therapy and behavioral therapy^8,10,11^.

This sample used the following sialorrhea management strategies: speech-language-hearing therapy, behavioral therapy, botulinum toxin injection, and surgical intervention.

The least invasive therapeutic interventions in our sample were speech and language therapy and behavioral therapy. Considering that sialorrhea results from poor oral control and inefficient swallowing^10^, improving this function is supposed to positively impact sialorrhea management. In this way, speech and language therapy is one of the first intervention options to manage sialorrhea.

In speech and language therapy, stimuli are used to adjust orofacial muscle tone and improve intraoral sensitivity, as well as oral motor exercises and chewing and swallowing training^8,10,11^.

Inal et al.^10^ showed a significant decrease in the severity scale for the group treated with functional chewing training, improving tongue movement and consequently swallowing; however, the comparison between the groups did not show a significant difference.

Some resources are generally used as support in speech-language-hearing therapy. A study^8^ investigated the efficacy of kinesio taping to help manage sialorrhea. It was proved to be effective, especially when used in combination with oral motor exercises.

Sethy and Mokashi^11^ investigated the effectiveness of conventional speech and language therapy and behavioral therapy. The results showed that behavioral therapy is effective when combined with conventional therapy, as children must have motor skills in order to swallow. Moreover, children must have preserved cognition to understand the rules, follow commands, and thus benefit from this strategy^11^.

Botulinum toxin injection into salivary glands was another therapeutic strategy contemplated in our sample. It is used as a strategy when conservative therapies do not control sialorrhea^13^. Considering that parotid and submandibular glands are responsible for producing the greatest volume of saliva, they are targeted in botulinum toxin intervention^7^. Studies indicate that this procedure is safe and effective to control sialorrhea^7,21^. However, they also highlight some side effects such as thickened saliva, xerostomia, and worsened swallowing function^7^.

Finally, surgical intervention is the last resource because it is the most invasive strategy. The literature describes various surgical techniques such as salivary gland resection and submandibular duct relocation. Research in the sample compared the effect of this surgery with botulinum toxin application. Results indicate a greater efficacy of the surgical procedure in question, but they call attention to the risks involved in surgery, even if they are minimal^13^.

Considering all sialorrhea management strategies, the individuality of each condition stands out. The strategy to be used must be decided by a multiprofessional team based on careful assessment and analysis of a variety of information, such as comorbidities, responses to other treatments, and the risk and benefit of each intervention. Moreover, combining therapies may be feasible and help avoid more invasive procedures^8,10,11^.

The evidence of outcomes ranged from moderate to very low, as there were limitations, inconsistencies, and imprecisions, e.g., not describing how randomization, allocation, and blinding were made. Some of them justified non-feasible blinding due to the different procedures being compared; also, most articles had significant drop-outs.

Interventions generally indicate decreased sialorrhea in the outcomes. However, in intragroup comparison, these variations were significant only regarding botulinum toxin^7^, oral motor exercises and kinesio taping combined with oral motor exercises^8^, and behavioral therapy^11^ in combination with conventional therapy. These results suggest the possibility of positive effects of such interventions; however, in comparison between groups, only the research comparing botulinum toxin with surgery^13^ presented significant differences between the groups, as surgery controlled sialorrhea more effectively.

Some methodological limitations must be considered. First, different sialorrhea assessment methods were used, and even though the Drooling Severity and Frequency Scale was used in four out of the five articles, they presented the results differently. Moreover, confounding factors, such as the severity of neurological disorders, may have influenced estimates, as few studies were included, while most of them were removed because of the study design or participants’ ages. Also, given the few articles in the sample and their heterogeneous methodology, it was not possible to conduct a meta-analysis.

## CONCLUSION

The studies that comprised the sample reported different interventions and outcome assessments. Considering the heterogeneous designs and the methodological limitations that impact the quality of evidence, the efficacy of the interventions could not be verified. However, most of them reported positive effects.
